# A clinical classification acknowledging neuropsychiatric and cognitive impairment in Huntington’s disease

**DOI:** 10.1186/s13023-014-0114-8

**Published:** 2014-07-17

**Authors:** Tua Vinther-Jensen, Ida U Larsen, Lena E Hjermind, Esben Budtz-Jørgensen, Troels T Nielsen, Anne Nørremølle, Jørgen E Nielsen, Asmus Vogel

**Affiliations:** 1 Neurogenetics Clinic, Danish Dementia Research Centre, Department of Neurology, Rigshospitalet, University of Copenhagen, Section 6922, Blegdamsvej 9, Copenhagen, DK-2100, Denmark; 2 Department of Cellular and Molecular Medicine, Section of Neurogenetics, University of Copenhagen, Copenhagen, Denmark; 3 Department of Psychology, University of Copenhagen, Copenhagen, Denmark; 4 Department of Biostatistics, University of Copenhagen, Copenhagen, Denmark

**Keywords:** Huntington’s disease, Clinical classification, Neuropsychiatry, Cognitive impairment, Premanifest

## Abstract

**Background:**

Involuntary movements, neuropsychiatric symptoms, and cognitive impairment are all part of the symptom triad in Huntington’s disease (HD). Despite the fact that neuropsychiatric symptoms and cognitive decline may be early manifestations of HD, the clinical diagnosis is conventionally based on the presence of involuntary movements and a positive genetic test for the HD CAG repeat expansion. After investigating the frequencies of the triad manifestations in a large outpatient clinical cohort of HD gene-expansion carriers, we propose a new clinical classification.

**Methods:**

In this cross-sectional study, 107 gene-expansion carriers from a Danish outpatient clinic were recruited. All participants underwent neurological examination, psychiatric evaluation and neuropsychological testing. Participants were categorised according to motor symptoms, neuropsychiatric symptoms, the use of psychotropic medication, and cognitive impairment.

**Results:**

Among the motor manifest HD gene-expansion carriers, 51.8% presented with the full symptom triad, 25.0% were defined as cognitively impaired in addition to motor symptoms, and 14.3% had neuropsychiatric symptoms along with motor symptoms. Only 8.9% had isolated motor symptoms. Among gene-expansion carriers without motor symptoms, 39.2% had neuropsychiatric symptoms, were cognitively impaired, or had a combination of the two.

**Conclusion:**

This is the first study to report the frequencies of both motor symptoms, cognitive impairment, and neuropsychiatric symptoms in HD gene-expansion carriers in a national outpatient HD clinical cohort. We found that almost 40% of the gene-expansion carriers without motor symptoms had either neuropsychiatric symptoms, cognitive impairment or both, emphasising that these patients are not premanifest in psychiatric and cognitive terms, suggesting that the current clinical classification is neither necessarily suitable nor helpful for this patient group. Some premanifest gene-expansion carriers may have psychiatric and/or cognitive symptoms caused by reactive stress or other pathology than HD. Acknowledging this fact we, however, suggest classifying all HD gene-expansion carriers into three clinical categories: *premanifest*, *non-motor manifest*, and *motor manifest*.

## Introduction

Huntington’s disease (HD) is an autosomal dominant neurodegenerative disorder presenting with progressive motor, cognitive, and neuropsychiatric symptoms [[1]]. The disease is caused by an expanded CAG repeat in the Huntingtin gene [[2]].

Since the first descriptions of HD it has been known that both “insanity” and “deterioration of the mind” in combination with chorea are part of the disease spectrum [[3]]. Currently, the clinical diagnosis is still based on unequivocal motor signs. For more than a decade, however, it has been acknowledged that in some cases the psychiatric and/or cognitive symptoms occur early in HD and may precede the motor symptoms by several years [[4]-[6]]. Moreover, for some patients, the psychiatric and cognitive symptoms tend to have a greater impact on maintaining everyday functioning and quality of life than the motor symptoms do [[7]]. The inadequacy of the motor criterion for clinical diagnosis of HD has recently been addressed by Loy and McCusker. They demonstrated, from a series of cases, the shortcomings of this motor-only diagnostic approach, arguing that both cognitive and psychiatric symptoms should be taken into account when giving a person the clinical diagnosis of HD [[8]].

The psychiatric symptoms found in HD are primarily irritability, depression, anxiety, and apathy [[9]]. The symptoms are assumed to be caused by the pathophysiological changes occurring in HD and not the awareness that a devastating disease will develop [[4],[6],[10]]. The manifestations and prevalence of psychiatric symptoms in HD gene-expansion carriers without motor symptoms have been questioned and discussed for over a decade [[4],[9]-[11]]. Two recent large multi-site longitudinal prospective studies designed to identify and track markers of HD prior to the onset of motor symptoms (PREDICT-HD and TRACK-HD) showed that both premanifest and manifest HD gene-expansion carriers revealed more psychiatric symptoms than healthy controls [[12],[13]].

Cognition has also been studied intensively in HD in the last ten years. Cognitive impairments are predominantly found in attention, executive function, and in psychomotor speed [[14],[15]]. In the PREDICT-HD study, “prodromal” HD gene-expansion carrier performance on cognitive test was dependent on proximity to diagnosis; individuals with < 9 years to diagnosis performed significantly worse than controls on 40 out of 51 tests [[16]]. In another paper form the PREDICT-HD study, nearly 40% of so-called “prediagnosed” HD gene-expansion carriers had a level of cognitive impairment that corresponded to the definition of mild cognitive impairment (MCI) [[17]]. In the TRACK-HD study, premanifest HD mutation carriers close to a clinical HD diagnosis showed a decline in cognition during a three-year period [[18]].

In previous studies on cognitive deficits and psychiatric symptoms, focus has been on the identification of sensitive measures of symptoms, and the large majority of studies have used comparisons at a group level to assess differences with regard to these symptoms [[13],[16],[19],[20]]. Other studies have focused on finding tests, scales or symptoms that could predict disease progression and/or phenoconversion [[16],[18]]. The frequency of cognitive deficits and psychiatric symptoms per se has, to our knowledge, never been investigated in a single large national cohort of manifest and premanifest HD gene-expansion carriers. Consequently, the objective of the present study is to investigate the frequency of motor symptoms, cognitive impairment, and psychiatric symptoms in a large group of HD gene-expansion carriers from a Danish HD outpatient clinic. Based on our results we propose a new clinical classification.

## Methods

### Subjects

Participants were recruited from January 2012 to March 2013 from the Neurogenetics Clinic, Danish Dementia Research Centre, Rigshospitalet, Copenhagen, Denmark. At the time of recruitment this clinic was the only specialised HD clinic in Denmark that general practitioners, clinical genetics departments, neurological departments or any other hospital department in the country referred HD gene-expansion carriers to, irrespective of being symptomatic or not. All individuals with a CAG repeat ≥39 and a Unified Huntington’s Disease Rating Scale-99, (UHDRS-motor) [[21]] total motor score ≤55, a Mini Mental State Examination (MMSE) score ≥24, and a Montreal Cognitive Assessment (MoCA) [[22]] score ≥19 were eligible for inclusion. Exclusion criteria were ongoing alcohol or drug abuse, and having a native language other than Danish. All individuals had been through a genetic counselling process and informed of their genetic status prior to participating in the study. The study was approved by the Ethics Committee of the Capital Region of Denmark (H2-2011-085), and written informed consent was obtained from each participant before enrolment.

All participants had a minimum of two planned visits. At the first visit the following examinations were performed: physical and neurological examination and neuropsychiatric evaluation. Neuropsychological testing was performed at the second visit. The two visits were performed in random order and the evaluations were performed blinded to one another. The same physician and neuropsychologist performed all examinations. A group of forty healthy HD gene-expansion negative individuals who were offspring of a HD gene-expansion carrier (and had been genetically tested with a CAG repeat length of less than 30) were included as controls to ensure the validity of the criteria for neuropsychiatric and cognitive assessments.

### Clinical evaluation

UHDRS-motor was applied to evaluate motor signs. Participants with a UHDRS-motor score of >5 were classified as motor manifest HD gene-expansion carriers. If the score was ≤5, indicating no substantial motor signs, they were classified as premanifest HD gene-expansion carriers*.* Furthermore, the subjects were assessed with UHDRS Total Functional Capacity (TFC) and UHDRS Function scales. Previous medical and psychiatric history was investigated and current medication noted. MoCA and MMSE were applied as cognitive screening instruments to exclude patients with cognitive impairment to a degree where neuropsychological testing would be unrewarding.

### Neuropsychiatric assessment and classification

To assess neuropsychiatric symptoms, the Symptom Checklist-90-Revised (SCL-90-R) [[23]] and the Hamilton Rating Scale for Depression-17 (HAM-17) were administered [[24]]. The SCL-90-R is a 90-item self-report symptom inventory designed to reflect the status of current psychological symptoms [[23]]. Each of the 90 items is rated on a five-point Likert scale of distress, ranging from “not at all” to “extremely”. Subsequently the questions and answers are divided into nine primary symptom dimensions: somatization (SOM), obsessive-compulsive (O-C), interpersonal sensitivity (I-S), depression (DEP), anxiety (ANX), hostility (HOS), phobic anxiety (PHOB), paranoid ideation (PAR), and psychoticism (PSY). The SCL-90-R also yields three global indices of distress: Global Severity Index (GSI), Positive Symptoms Distress Index (PSDI), and Positive Symptoms Total (PST) [[23]]. Raw scores can be converted to T-scores normalised to a Danish sample of non-psychiatric individuals sorted by gender [[25]]. Higher T-scores indicate more psychiatric distress.

The investigator administered the HAM-17 in a semi-structured interview covering the 17 symptom areas.

Participants were allocated to the neuropsychiatric group based on at least one of the following criteria:

1. Usage of psychotropic medication

2. A SCL-90-R GSI T-score ≥63 or a T-score ≥63 in more than two of the nine primary symptom dimensions. The SCL-90-R cut-offs are based on SCL-90-R guide [[23],[26]]

3. A HAM-17 score ≥13 (moderate to severe depression) [[27]]

### Neuropsychological testing and classification

*Pre-morbid intellectual level* was assessed by the Wechsler Adult Intelligence Scale (WAIS) Vocabulary subtest and the Danish Adult Reading Test (DART), a Danish equivalent of the National Adult Reading Test [[28]]. *Memory* was assessed with the Selective Reminding Test, both immediate recall (errors were recorded) and delayed recall (retention interval 10 min) [[29]] and the Rey Complex Figure Test (recall 3 min) [[30]]. *Psychomotor speed/Attention* was assessed by Trail Making Test A & B [[31]] (only completion time used for analyses) and Symbol Digit Modalities Test [[32]]. *Executive functions* were assessed with the Stroop test (100 items) [[33]] and verbal fluency tests. For the Stroop test, only performance on the incongruent version was used for analyses (only completion time was used). We applied three verbal fluency tests*:* category fluency (animals, 1 min) and lexical fluency (s-words and a-words, 1 min); these measures were analysed separately. *Visuospatial functions* were assessed using a Rey Complex Figure [[30]], Ravens Progressive Matrices (set 1) [[34]], and a modified version of the Block Design Test [[35]].

The normative data for the neuropsychological tests used in this study were derived from the test results from 80 age-matched healthy subjects, retrieved from a database at the Department of Neurology, Rigshospitalet, University of Copenhagen. For each test, expected scores were generated from factors based on regression analyses including age, years of education and general verbal intellectual level (as assed by the Vocabulary subtest from WAIS and DART). To assess if observed scores differed from expected scores and could be categorised as impaired, the variation in residual values from the regression analyses was used. Difference scores between observed and expected scores were used to evaluate impairment [[36]]. Scores above the tenth percentile of the normal variation in the regression analyses were categorised as unimpaired, whereas difference scores in the lowest 10% of the normal variation were categorised as impaired.

The following criteria for classifying a patient as cognitively impaired were applied: a) if four (or more) test performances were categorised as impaired; b) if all test performances in a domain (except psychomotor speed/attention) were impaired; c) if performances on all tests in the psychomotor speed/attention domain, and if at least one other test, were below the cut-off.

### Data analysis

Participants were classified into four groups based on the presence of cognitive impairment and neuropsychiatric symptoms. The relative frequency of the groups was estimated for manifest and pre-manifest subjects. Using logistic regression analysis, we then explored whether demographic and clinical variables predicted the symptom dimensions. In the manifest group, we modelled the risk of having both cognitive impairment and neuropsychiatric symptoms as a function of relevant covariates, including gender, CAG repeat length, and disease duration. For the premanifest group we modelled the risk of having either cognitive impairment, neuropsychiatric symptoms, or both as a function of the covariates mentioned above and the disease burden score ((CAG_n_ – 35.5)*Age) [[37]]. The covariates were initially included one at a time, and then a model including all covariates was developed. Effects are presented as odds ratios (OR).

In addition, we compared the neuropsychological and neuropsychiatric test scores between manifest and premanifest subjects. These outcomes approximately followed normal distributions, and the comparison was therefore based on an independent sample t-test. A *p*-value of less than 0.05 was considered significant.

## Results

One hundred and thirty-four gene-expansion carriers were asked to participate; 12 were excluded due to either low scores on MoCA or MMSE, and/or high scores on UHDRS motor. Fifteen did not meet on arranged trail dates or declined to participate in the study.

One hundred and seven participants fulfilled the inclusion criteria and completed the study programme, 51 were premanifest gene-expansion carriers according to the motor criterion and the remaining 56 were motor manifest. Table 1 contains clinical data from the two groups. There was no significant difference in gender or CAG repeat length in the two groups, but the motor manifest group, as expected, was significantly older and had higher UHDRS motor and lower TFC scores than the premanifest group. In the premanifest group the majority (>90%) completed the genetic counseling and testing procedure more than one year prior to inclusion in this study.

**Table 1:** Clinical characteristics of the two groups of participants

	***Premanifest HD gene-expansion carriers***	***Motor manifest HD gene-expansion carriers***	***Level of significance***
	***N = 51***	***N = 56***	***p-values***
*Gender (m/f)*^*§*^	30/21	33/23	p = 0.32
*Age at examination*^#^*(years)*	36 (20–54)	50 (24–75)	p < 0.001
*CAG repeat length*^#^	42 (39–48)	43 (40–53)	p < 0.38
*Disease burden score*^*a*^	234.0 (108.0–437.0)		
*UHDRS-motor*^*b*^*(score)*^#^	2 (0–5)	21 (6–51)	p < 0.001
*TFC*^*c*^*(score)*^#^	13 (11–13)	10 (4–13)	p < 0.001
*MMSE (score)*^#^	29 (26–30)	28 (24–30)	p < 0.001
*MoCA (score)*^#^	28 (25–30)	25 (19–30)	p < 0.001

Data are presented as median (range).

p values were calculated by ^*§*^Chi-square-test or ^#^Mann–Whitney U test.

^a^Disease burden score = ((CAG_n_ – 35.5)*Age); ^b^UHDRS-motor: Unified Huntington’s Disease Rating Scale-99, range (0–124); ^c^TFC: Total Function Capacity, range (0–13).

There were no significant differences between the two groups on the HAM-17 rating scale or on the SOM, I-S, ANX, HOS, PHOB, and PAR SCL-90-R symptom dimensions, but on the O-C, DEP, PSY symptom dimensions and, on GSI, motor manifest gene-expansion carriers had significantly higher scores compared to the premanifest gene-expansion carriers (Table 2). After Bonferroni correction, these differences, except the O-C dimension, became insignificant. On all neuropsychological tests motor manifest HD gene-expansion carriers performed significantly worse as compared to premanifest HD gene-expansion carriers (Table 2).

**Table 2:** Neuropsychiatric symptoms and neuropsychological test performances in the premanifest and the motor manifest gene-expansion carriers

	***Premanifest HD gene-expansion carriers***	***Motor manifest HD gene-expansion carriers***	***Level of significance***
	***N = 51***	***N = 56***	***p-value***
**Neuropsychiatric scales**			
*SCL-90-R*^*a*^			
*Global Severity Index*	49.6 (10.1)	53.6(10.2)	0.041
*Somatization*	50.5 (9.7)	50.5 (9.9)	0.979
*Obsessive-compulsive*	49.5 (10.3)	57.25 (10.2)	<0.001
*Interpersonal Sensitivity*	47.9 (10.5)	51.2 (12.0)	0.134
*Depression*	49.3 (10.5)	54.0 (9.0)	0.014
*Anxiety*	50.1 (10.9)	53.0 (9.46)	0.141
*Anger-hostility*	53.3 (11.6)	52.8 (12.7)	0.848
*Phobic anxiety*	50.7 (11.1)	55.1 (12.8)	0.059
*Paranoid ideation*	47.4 (9.7)	47.2 (9.6)	0.915
*Psychoticism*	47.8 (9.2)	53.4 (10.9)	0.005
*Positive Symptoms Total*	49.9 (10.0)	52.6 (9.1)	0.142
*Positive Symptoms Distress Index*	52.6 (10.3)	55.7 (10.8)	0.141
*HAM-17*^*b*^	4.5 (4.6)	5.8 (3.7)	0.103
***Neuropsychological tests***			
*Trail Making Test A (seconds)*	22.8 (5.2)	45.3 (21.2)	<0.001
*Trail Making Test B (seconds)*	62.2 (16.7)	131.8 (55.5)	<0.001
*Symbol Digit Modalities Test (number correct)*	49.6 (8.1)	28.3 (9.4)	<0.001
*Stroop interference (seconds)*	119.0 (21.0)	193.5 (77.3)	<0.001
*Category verbal fluency*	24.1 (4.2)	16.6 (6.4)	<0.001
*Lexical fluency, s-words*	14.5 (3.9)	10.7 (5.1)	<0.001
*Lexical fluency, a-words*	9.4 (3.9)	5.7 (3.1)	<0.001
*Reys Complex Figure Test, recall*	24.3 (6.3)	18.3 (6.0)	<0.001
*Reys Complex Figure Test, copy*	35.6 (1.0)	34.0 (2.4)	<0.001
*Block design (number correct)*	11.9 (0.4)	10.8 (1.8)	<0.001
*Raven’s Advanced Progressive Matrices*	9.5 (1.7)	6.5 (2.6)	<0.001
*Selective Reminding Test, immediate recall (number of errors)*	12.49 (7.9)	28.17 (11.6)	<0.001
*Selective Reminding Test, delayed recall*	7.57 (1.7)	6.02 (2.1)	<0.001

^a^Symptom Checklist-90-R. Data shown as mean (SD). p values were calculated by independent sample t-tests. ^b^Hamilton Rating Scale for Depression-17.

The validity of the criteria for neuropsychiatric and cognitive assessment was tested by applying them to the group of 40 healthy gene-expansion negative individuals. Among these, two participants (5.0%) fulfilled the criteria for neuropsychiatric grouping and two participants (5.0%) were classified as cognitively impaired; one participant could be classified both with neuropsychiatric symptoms and as cognitively impaired.

### Neuropsychiatric classification

**Figure 1:**
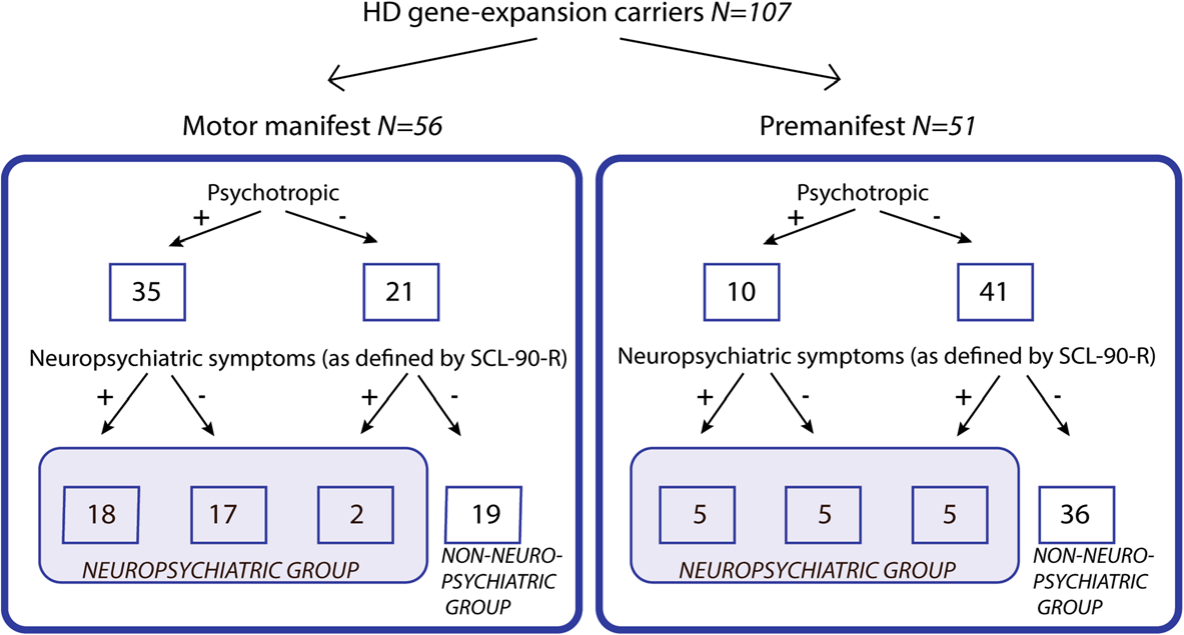
Flowchart used for categorisation of participants into the neuropsychiatric vs. the non-neuropsychiatric groups.

The diagram in Figure 1 shows the classification of the participants into the neuropsychiatric group and non-neuropsychiatric group. Among the premanifest gene-expansion carriers ten participants (19.6%) were treated with antidepressants and 51.1% of all participants taking antidepressants had neuropsychiatric symptoms according to the SCL-90-R. In the motor manifest group, 35 participants (61.0%) were treated with antidepressants; of these 51.4% had neuropsychiatric symptoms according to the SCL-90-R. Among participants without psychotropic medication seven, (five premanifest (9.8%) and two motor manifest (3.6%)) had neuropsychiatric symptoms according to the SCL-90-R criteria. Participants were taking different antidepressants (including SSRI, SNRI, and NaSSA). Two patients had antipsychotics alone: One was classified in the neuropsychiatric group based on the SCL-90-R score while the other had tetrabenazine solely for involuntary movements and scores below the cut-off in SCL-90-R. This patient was categorised as non-neuropsychiatric.

### Neuropsychological examination

The large majority of motor manifest gene-expansion carriers (N = 43, 76.8%) and a subset of the premanifest gene-expansion carriers (N = 7, 13.7%) were classified as cognitively impaired according to our criteria.

### Clinical characterisation of the HD gene-expansion carriers

**Figure 2:**
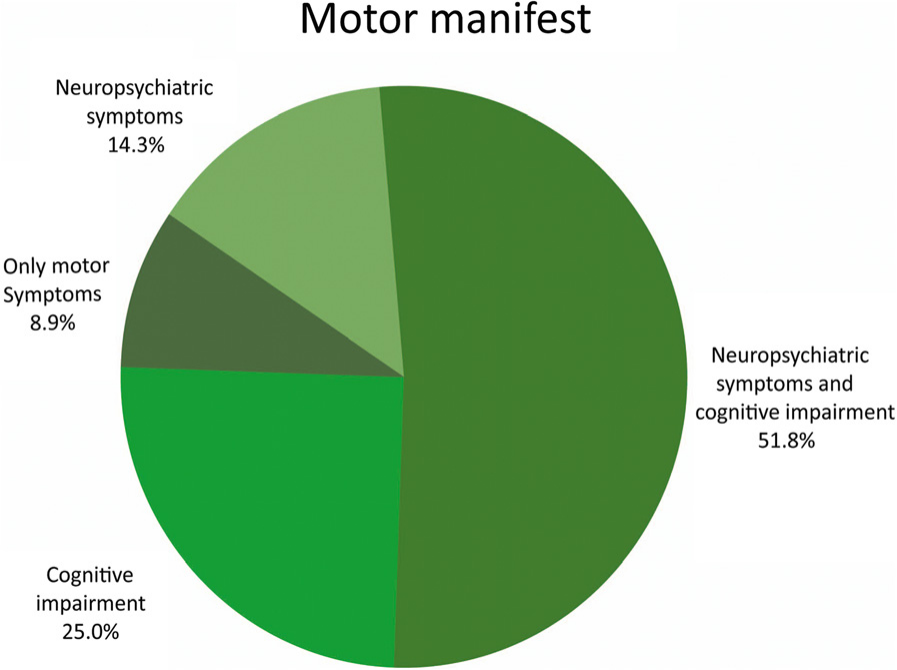
Distribution of cognitive impairment and neuropsychiatric symptoms in the motor manifest HD gene-expansion carriers.

Figure 2 shows the distribution of cognitive impairment and neuropsychiatric symptoms in the motor manifest group. Very few had isolated motor symptoms (8.9%), and more than half (51.8%) had a symptom complex with motor symptoms, neuropsychiatric symptoms, and cognitive impairment.

Covariates were not able to predict whether motor manifest gene-expansion carriers displayed the full triad of symptoms or not. Male gender, younger age, longer CAG repeat length, higher UHRDS score, and shorter disease duration were associated with an increased risk for having both neuropsychiatric symptoms and cognitive impairment, but none of these effects was statistically significant (data not shown). Effects remained insignificant when included simultaneously in a multiple logistic regression model. However, a decrease in TFC score was associated with a significant increased risk of symptoms (OR=0.50, p<0.001).

**Figure 3:**
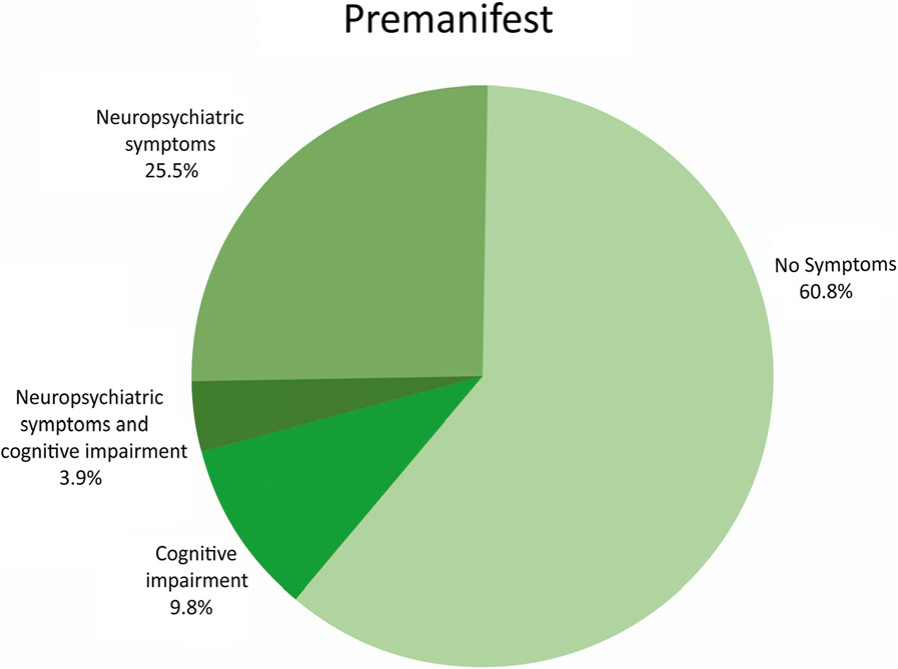
Distribution of cognitive impairment and neuropsychiatric symptoms in the premanifest HD gene-expansion carriers.

In the premanifest group, 60.8% were classified without neuropsychiatric symptoms and/or cognitive impairment (Figure 3), and 39.2% had neuropsychiatric symptoms, cognitive impairment or a combination of the two. Logistic regression models showed that male gender, older age, shorter CAG repeat length and higher disease burden predicted an increased risk of being in the group with cognitive impairment, neuropsychiatric symptoms, or both. However, all effects were far from reaching statistical significance. Effects remained insignificant when included simultaneously in a multiple regression model.

## Discussion

To our knowledge, this is the first study to investigate the frequency of both psychiatric symptoms and cognitive impairment in a national clinical cohort of premanifest to moderately affected HD gene-expansion carriers. In this cross-sectional study the triad of motor symptoms, neuropsychiatric symptoms, and cognitive impairment was found in 51.8% of all motor manifest HD gene-expansion carriers. Further, we showed that among the premanifest HD gene-expansion carriers, 39.2% had neuropsychiatric symptoms, cognitive impairment, or both.

In the motor manifest group, 76.8% of the patients fulfilled the criteria for cognitive impairment. The results show that cognitive deficits are found in the large majority of the motor manifest patients seen in the clinic (even in the early stages of HD). In addition, the results imply that cognitive evaluation is very important in HD motor manifest patients, since such deficits could have a negative effect on the patient’s ability to, for example, maintain a job, manage household finances, or drive a car.

In the premanifest group, 13.7% were classified as cognitively impaired. The premanifest gene-expansion carriers are generally considered to be healthy and are treated as such; however, the results show that neuropsychological assessment and follow-up may be relevant, e.g. to try to prevent financial stagnation and social exclusion. The frequency of cognitive impairment in HD has been investigated in a previous study, and the frequency of MCI in “prediagnosed” HD gene-expansion carriers was examined using standard criteria for MCI (test performance in one of four tests below 1.5 standard deviations on age- and education-adjusted normative data). MCI was found in 39.8% and multiple-domain MCI (two or more tests below 1.5 standard deviations) in 13.9% of the “prediagnosed” HD gene-expansion carriers [[17]]. The methodology of our study compared to the MCI study is different, and we used a more conservative classification of cognitive impairment and the premanifest status. Importantly, both studies show that cognitive impairment is present in some presymptomatic HD gene-expansion carriers. Our study further suggests that cognitive symptoms in premanifest HD are not only mild, but also that premanifest HD gene-expansion carriers may have cognitive deficits to an extent where they may be classified as cognitively impaired.

According to our predefined criteria, 66.1% in the motor manifest group and 29.4% in the premanifest group were classified to the neuropsychiatric group. Previous studies have reported very different lifetime prevalence of psychiatric symptoms in HD gene-expansion carriers that varies from 24.7% to 76.0% in manifest patients [[9]]. A recent study reported a 12-month prevalence of psychiatric symptoms of 24.7% in manifest and of 27.3% in premanifest HD gene-expansion carriers [[6]]. The frequencies in our study are markedly higher as compared to Van Duijn et al’s study [[6]]. We chose an all or none approach in our study. If the participants had an intake of psychotropic medication, they were classified in the neuropsychiatric group, without regard to their current psychiatric symptoms. Psychotropic medication is prescribed by physicians who have found indication for the treatment. Moreover, we assume that the participants taking psychotropic medication do so because they have an underlying psychiatric disorder and the majority of these patients would probably have affective or other psychiatric symptoms if their medication were paused. Therefore, not classifying patients on psychotropic medication in the neuropsychiatric group seems, in our opinion, counterintuitive. However, the frequency of psychiatric symptoms according to the SCL-90-R in our cohort was 35.7% in the motor manifest group and 19.6% in the premanifest group, which is in accordance with the Van Duijn et al’s study [[6]].

It is important to recognise that both psychiatric symptoms and/or cognitive dysfunction may be stress disorders, reactive to life events, and in the case of HD, a transient reaction to receiving the genetic test result [[38]], and therefore not necessarily a consequence of neurodegeneration. However, the majority of our premanifest participants completed the genetic counseling procedure more than one year prior to inclusion in this study, which makes a test-related reactive disorder less likely. The criteria for cognitive impairment in our study are consistent with severe impairment, and the cognitive deficits in depression in general are described as mild to moderate in group comparisons [[39]], a pattern which was equally found in prodromal HD in the PREDICT-HD study [[40]]. Therefore, cognitive impairment may not solely be attributed to depression or other psychiatric symptoms but a consequence of the neuropathological changes as well.

Antidepressants exert more positive than negative effects on cognitive dysfunction in patients with depression [[41]]. The effect of antidepressants on cognitive performance has not been studied in HD; however, it seems likely that treatment may also improve cognitive function due to depression in HD gene-expansion carriers.

When applying our predefined criteria of cognitive impairment and neuropsychiatric symptoms to a Danish control cohort of 40 healthy HD gene-expansion negative individuals, the frequency of cognitive impairment was, as expected, 5%. The frequency of psychiatric symptoms was, however, lower than the expected 10% [[26]], confirming that our criteria for cognitive impairment and neuropsychiatric symptoms are suitable and adequately conservative for use in the Danish population.

The patients included in this study all had an MMSE score ≥24, a MoCA score ≥19 and a UHDRS motor ≤55. These cut-off scores for inclusion were applied because patients with lower scores would not be capable of doing a meaningful neuropsychological testing in this study. This selection has important implications because the frequency of cognitive deficits and neuropsychiatric symptoms in this selected group of HD gene-expansion carriers cannot be generalised to the entire HD population. A small subgroup of invited participants either declined participation or did not meet for arranged trial dates. Evaluating the patient files of this patient group showed that the majority of these patients would have been classified in the neuropsychiatric group. We therefore hypothesise that both cognitive impairments and psychiatric symptoms are more frequent in the “general” HD population.

Despite these limitations, we demonstrate the importance of the assessment of all HD gene-expansion carriers with motor, neuropsychiatric, and cognitive measures regularly to ensure sufficient and early treatment and social intervention.

## Conclusion

Almost 40% of the gene-expansion carriers without motor symptoms had either neuropsychiatric symptoms, cognitive impairment or both, emphasising that these patients are not premanifest in psychiatric and cognitive terms. Currently, the clinical diagnosis of HD relies on motor symptoms; however, both cognitive impairment and neuropsychiatric symptoms may be very early and substantial signs, and as it is impossible to predict the symptom complex of premanifest gene-expansion carriers, we recommend that all HD gene-expansion carriers are referred to/offered a regular follow-up programme that includes a baseline neuropsychological and neuropsychiatric evaluation as early as possible. This is further underlined by the fact that the large negative impact that psychiatric illness has on the quality of life and the major burden that it places on relatives [[42]] can be alleviated or potentially eliminated. Therefore, screening for psychiatric symptoms in HD has important perspectives. Furthermore, to acknowledge the high proportion of gene-expansion carriers who, based on the lack of motor symptoms, traditionally are classified as “premanifest”, but who have psychiatric symptoms, cognitive deficits, or both, we propose a new clinical classification into three different groups: *premanifest*, without symptoms or signs within the triad spectrum; *non-motor-manifest*, with neuropsychiatric and/or cognitive impairment, but without motor signs; and *motor-manifest*, with motor signs, and possible neuropsychiatric and/or cognitive impairment. Our predefined criteria for neuropsychiatric and cognitive impairment, however, will have to be elaborated and validated further in larger cohorts.

It is important to recognise that both psychiatric and/or cognitive symptoms may be stress-related disorders and therefore e.g. a depression may stand alone as a diagnosis in premanifest HD subjects, without fulfilling the criteria for non-motor manifest.

The suggested classification will make a diagnosis of manifest HD possible on neuropsychiatric and/or cognitive impairment alone, and thereby ensure early and relevant treatment and psycho-social intervention for the benefit of the patients, relatives, and caregivers.

## Competing interests

The authors declare that they have no competing interests.

## Authors’ contributions

All authors have been involved in the planning and conduction of the study, as well as the analysis and interpretation of data. TVJ and IUA have primarily been responsible for the clinical data. All authors have been engaged in drafting the article and revising it critically for important intellectual content. All authors have approved the submitted version.
